# Human Epidermal Growth Factor Receptor 2 (HER2) in Cancers: Overexpression and Therapeutic Implications

**DOI:** 10.1155/2014/852748

**Published:** 2014-09-07

**Authors:** Nida Iqbal, Naveed Iqbal

**Affiliations:** ^1^Department of Medical Oncology, Dr. B. R. A. Institute Rotary Cancer Hospital, All India Institute of Medical Sciences, New Delhi 110029, India; ^2^Department of Anaesthesia and Intensive Care Unit, Indraprastha Apollo Hospital, New Delhi 110076, India

## Abstract

Human epidermal growth factor receptor 2 (HER2) is a member of the epidermal growth factor receptor family having tyrosine kinase activity. Dimerization of the receptor results in the autophosphorylation of tyrosine residues within the cytoplasmic domain of the receptors and initiates a variety of signaling pathways leading to cell proliferation and tumorigenesis. Amplification or overexpression of HER2 occurs in approximately 15–30% of breast cancers and 10–30% of gastric/gastroesophageal cancers and serves as a prognostic and predictive biomarker. HER2 overexpression has also been seen in other cancers like ovary, endometrium, bladder, lung, colon, and head and neck. The introduction of HER2 directed therapies has dramatically influenced the outcome of patients with HER2 positive breast and gastric/gastroesophageal cancers; however, the results have been proved disappointing in other HER2 overexpressing cancers. This review discusses the role of HER2 in various cancers and therapeutic modalities available targeting HER2.

## 1. Introduction

The human epidermal growth factor receptor (HER) family of receptors plays a central role in the pathogenesis of several human cancers. They regulate cell growth, survival, and differentiation via multiple signal transduction pathways and participate in cellular proliferation and differentiation. The family is made up of four main members: HER-1, HER-2, HER-3, and HER-4, also called ErbB1, ErbB2, ErbB3, and ErbB4, respectively [1]. All four HER receptors comprise a cysteine-rich extracellular ligand binding site, a transmembrane lipophilic segment, and an intracellular domain with tyrosine kinase catalytic activity [2]. Epidermal growth factor receptor (EGFR, ErbB1, and HER1)—the first receptor tyrosine kinase, was discovered by Carpenter and coworkers at Vanderbilt University, USA, in 1978 [3]. ErbB stands for its origin in the Erb-b gene responsible for avian erythroblastosis virus. The neu oncogene (also known as HER2, ErbB2, or p185) was discovered by a group of scientists at Massachusetts Institute of Technology, Rockefeller, and Harvard University [4, 5]. The HER2 receptor is a 1255 amino acid, 185 kD transmembrane glycoprotein located at the long arm of human chromosome 17 (17q12) [6]. HER2 is expressed in many tissues and its major role in these tissues is to facilitate excessive/uncontrolled cell growth and tumorigenesis [7–9].

## 2. Function

The HER receptors exist as monomers on the cell surface. Upon ligands binding to their extracellular domains, HER proteins undergo dimerization and transphosphorylation of their intracellular domains. HER2 has no known direct activating ligand and may be in an activated state constitutively or become active upon heterodimerization with other family members such as HER1 and HER3. Homo- or heterodimerization results in the autophosphorylation of tyrosine residues within the cytoplasmic domain of the receptors and initiates a variety of signaling pathways, principally the mitogen-activated protein kinase (MAPK), phosphatidylinositol-4,5-bisphosphate 3-kinase (PI3K), and protein kinase C (PKC) resulting in cell proliferation, survival, differentiation, angiogenesis, and invasion. Heterodimers generate more potent signals than homodimers, and those containing HER2 have a particularly high ligand binding and signaling potency as HER2 exists in an open conformation making it the dimerization partner of choice among the family members. The HER2-HER3 heterodimer is the most potent stimulator of downstream pathways, particularly the PI3K/Akt, a master regulator of cell growth and survival. Moreover, HER2 dimerization promotes the mislocalization and rapid degradation of cell-cycle inhibitor p27^Kip1^ protein leading to cell-cycle progression [7, 10, 11]. HER2 can also be activated by complexing with other membrane receptors such as insulin-like growth factor receptor 1 [12].

**Figure 1:**
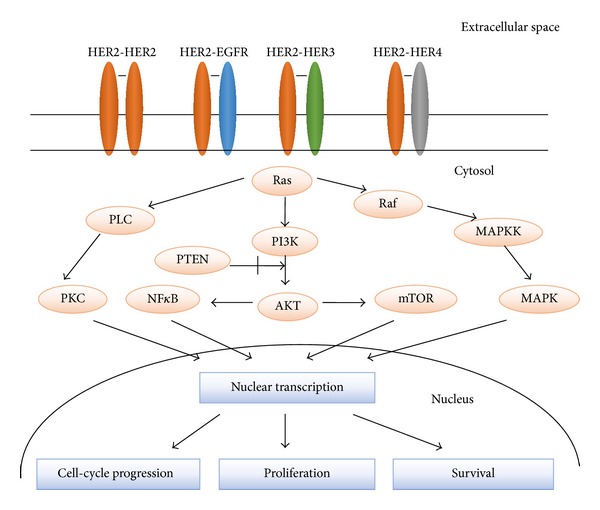
Receptor homodimerization or heterodimerization leads to activation of downstream signaling pathways promoting cell growth, proliferation, and survival. HER2 exists in an open conformation making it the dimerization partner of choice among the family members. The PI3K/AKT axis (which is regulated by PTEN and involves other key effectors such as NF*κ*B and mTOR) and the Raf/MAPK cascade are the two most important and most extensively studied downstream signaling pathways that are activated by the HER receptors. Ras is at the top of these cascades and acts as a self-inactivating signal transducer. A third important factor in the network is PKC, which is activated by PLC. As a result of these signaling pathways, different nuclear factors are recruited and modulate the transcription of different genes involved in cell-cycle progression, proliferation, and survival. EGFR, epidermal growth factor receptor; HER, human epidermal growth factor receptor; PLC, phospholipase C; PKC, protein kinase C; PI3K, phosphatidylinositol 3-kinase; PTEN, phosphatase and tensin homolog; NF*κ*B, nuclear factor *κ*B; mTOR, mammalian target of rapamycin; MAPK, mitogen-activated protein kinase; MAPKK, MAPK kinase.

Figure 1 [13] shows the main transduction pathways regulated by the four HER family members—EGFR, HER2, HER3, and HER4.

## 3. HER2 Overexpression in Cancers

Most of the studies on HER2 have been carried out in breast cancer, after it was found to induce mammary carcinogenesis in vitro [14] and in vivo [15]. Amplification or overexpression of the HER2 gene occurs in approximately 15–30% of breast cancers [16]. With increasing understanding of HER2 biology, it has now been recognized that HER2 overexpression occurs in other forms of cancers also such as stomach, ovary, uterine serous endometrial carcinoma, colon, bladder, lung, uterine cervix, head and neck, and esophagus [17, 18]. Apart from its role in development of various cancers, it has also been intensely evaluated as a therapeutic target. The aim of this review is to update the role of HER2 in various cancers.

### 3.1. HER2 in Breast Cancer

HER2 is overexpressed in 15–30% of invasive breast cancers, which has both prognostic and predictive implications [16]. Breast cancers can have up to 25–50 copies of the HER2 gene, and up to 40–100-fold increase in HER2 protein resulting in 2 million receptors expressed at the tumor cell surface [19]. Even estrogen, working via the nongenomic activity of estrogen receptor (ER) outside the nucleus, has been shown to activate HER2 signaling [20]. An aberrant form of HER2 (known as p95), lacking the extracellular domain, is found in some breast cancers. p95 is constitutively active and causes resistance to trastuzumab which requires the extracellular domain of HER2 for binding. For the same reason, p95 is not detected by antibodies that target the extracellular domain [21, 22].

HER2 gene amplification is associated with shorter disease-free and overall survival in breast cancer. Slamon et al. [23] established the prognostic significance of HER2 amplification in 189 human breast cancers. Amplification of HER2 gene was found to be a significant predictor of both overall survival (*P* < 0.001) and time to relapse (*P* < 0.0001). In a study by Press et al. [24], the expression of HER2 was studied in 704 node-negative breast cancers and it was found that women with breast cancer having high overexpression had a risk of recurrence 9.5 times greater than those whose breast cancers had normal expression (*P* = 0.0001). Analysis of various subgroups showed that the increased risk of recurrence extended across several subgroups of node-negative breast cancer patients. Seshadri et al. [25] in their study of 1056 patients with Stages I–III breast cancer found that HER2 amplification 3-fold or greater was associated with significantly shorter disease-free survival (*P* = 0.0027). HER2 amplification also correlated significantly with pathologic stage of disease, number of axillary nodes with tumor, histologic type, and absence of estrogen receptor (ER) and progesterone receptor (PgR). Evidence suggests that HER2 amplification is an early event in human breast tumorigenesis. HER2 amplification is seen in nearly half of all in situ ductal carcinomas without any evidence of invasive disease and HER2 status is maintained during progression to invasive disease, nodal metastasis, and distant metastasis [26]. HER2 amplified breast cancers have increased sensitivity to certain cytotoxic chemotherapeutic agents and resistance to certain hormonal agents and increased propensity to metastasize to the brain [27].

### 3.2. HER2 in Gastric Cancer

HER2 overexpression in patients with gastric cancer has been reported from 10 to 30% and correlates with poor outcome and a more aggressive disease. Overexpression of HER2 protein in gastric cancer, using immunohistochemistry (IHC), was first described in 1986 [28]. In a study by Yano et al. [29], HER2 overexpression by IHC was found in 23% and gene amplification by FISH in 27% of 200 resected tumors. Gravalos and Jimeno [30] in their study of 166 gastric cancer patients observed that HER2 overexpression was most commonly found in gastroesophageal junction (GEJ) tumors and tumors having intestinal type histology. Other studies also confirmed a higher rate of HER2 positivity in GEJ tumors and intestinal subtype [31, 32]. HER2 overexpression is directly correlated with poorer outcome in gastric cancer. In a study of 260 gastric cancers, HER2 overexpression was an independent negative prognostic factor and HER2 staining intensity was correlated with tumor size, serosal invasion, and lymph node metastases [33]. Other studies also confirmed the negative impact of HER2 overexpression in gastric cancer [34, 35].

### 3.3. HER2 in Esophageal Cancer

HER2 overexpression is reported in 0–83% of esophageal cancers, with a tendency towards higher rates of positivity in adenocarcinoma (10–83%) compared to squamous cell carcinomas (0–56%) [36–39]. Yoon et al. [40], in their study of 713 patients with surgically resected esophageal adenocarcinomas (EAC), found HER2 positivity in 17% patients and it was significantly associated with lower tumor grade, less invasiveness, fewer malignant nodes, and the presence of adjacent Barrett's esophagus (BE). In EACs with Barrett's esophagus (BE), HER2 positivity was significantly associated with improved DSS [HR = 0.54 (95% CI: 0.35–0.84), *P* = 0.0065] and overall survival (*P* = 0.0022) independent of pathologic features but was not prognostic among EACs without BE. However, another study by the same authors found that HER2 heterogeneity among HER2 amplified EACs was an independent predictor of worse cancer-specific survival [41]. Apart from EAC, HER2 overexpression was also found to be a negative predictor of survival in esophageal squamous cell carcinoma [42].

### 3.4. HER2 in Ovarian Cancer

Overexpression of HER2 is seen in 20–30% patients with ovarian cancer. Association of HER2 overexpression with poor survival in advanced epithelial ovarian cancer was first established by Berchuck et al. [43]. In a cohort of 73 patients with ovarian cancer, patients with HER2 overexpression had significantly worse survival as compared to patients with normal expression. In addition, patients whose tumors had high HER2 expression were significantly less likely to have a complete response to primary therapy or have a negative second-look laparotomy when serum CA 125 levels were normal preoperatively. Bartlett et al. [44] in their study of 76 patients with ovarian malignancy found that patients with tumors possessing EGF receptor mRNA had significantly reduced survival as compared to patients with tumors having negative expression. Although HER2 overexpression has been found to be associated with poorer survival, the usefulness of HER2 directed therapies is limited due to low frequency of strong expression.

### 3.5. HER2 in Endometrial Cancer

In endometrial serous carcinoma, the reported rates of HER2 overexpression range between 14% and 80% with HER2 amplification (by fluorescence in situ hybridization [FISH]) ranging from 21% to 47%. HER2 overexpression and amplification in endometrioid carcinomas have been reported in the range from 1% to 47% and from 0% to 38%, respectively [45–47]. Both HER2 overexpression and amplification have been linked to poor prognosis in endometrial carcinoma. Santin et al. [48] reported dramatically shorter overall survival in patients with HER2-amplified endometrial serous carcinoma compared with those without amplification. In addition, patients with high HER2 copy numbers (ratio >2.5) did significantly worse than those with lower HER2 amplification (ratio 2.0–2.5).

*HER2 in Other Cancers*. In lung cancers, overexpression of HER2 has been reported in about 20% [49, 50]. Apart from overexpression, mutations of HER2 were also reported in lung adenocarcinomas. The mutations targeted never or light smokers, oriental ethnicity, and female gender [51, 52]. In invasive urothelial bladder carcinomas, amplification and/or overexpression range from 23% to 80% for overexpression and from 0% to 32% for amplification [53, 54]. However, clinical trials using HER2 directed therapies in lung and bladder cancers reported disappointing clinical benefits [55, 56].

## 4. Testing for HER2

Although several methods for HER2 testing have been developed, approximately 20% of current HER2 testing may be inaccurate. Therefore, the American Society of Clinical Oncology (ASCO) and the College of American Pathologists (CAP) have recommended guidelines in HER2 testing to ensure accuracy [57]. The two methods currently approved for HER2 testing are immunohistochemistry (IHC) and fluorescence in situ hybridization (FISH).

*Breast Cancer*. HER2 status should be determined in all patients with invasive breast cancer on the basis of 1 or more test results. Breast cancer specimens should initially undergo HER2 testing by a validated immunohistochemistry (IHC) assay for HER2 protein expression [58]. The scoring method for HER2 expression is based on the cell membrane staining pattern and is as follows:

- 3+: positive HER2 expression, uniform intense membrane staining of more than 30% of invasive tumor cells;
- 2+: equivocal for HER2 protein expression, complete membrane staining that is either nonuniform or weak in intensity but has circumferential distribution in at least 10% of cells;
- 0 or 1+: negative for HER2 protein expression.

Breast cancer specimens with equivocal IHC should undergo validation fluorescence in situ hybridization (FISH). The interpretation for HER2 FISH testing (HER2-to-CEP17 ratio and gene copy number) is as follows:

- positive* HER2* amplification: FISH ratio higher than 2.2 or* HER2* gene copy greater than 6.0;
- equivocal* HER2* amplification: FISH ratio of 1.8–2.2 or* HER2* gene copy of 4.0–6.0;
- negative* HER2* amplification: FISH ratio lower than 1.8 or* HER2* gene copy less than 4.0.

**Figure 2:**
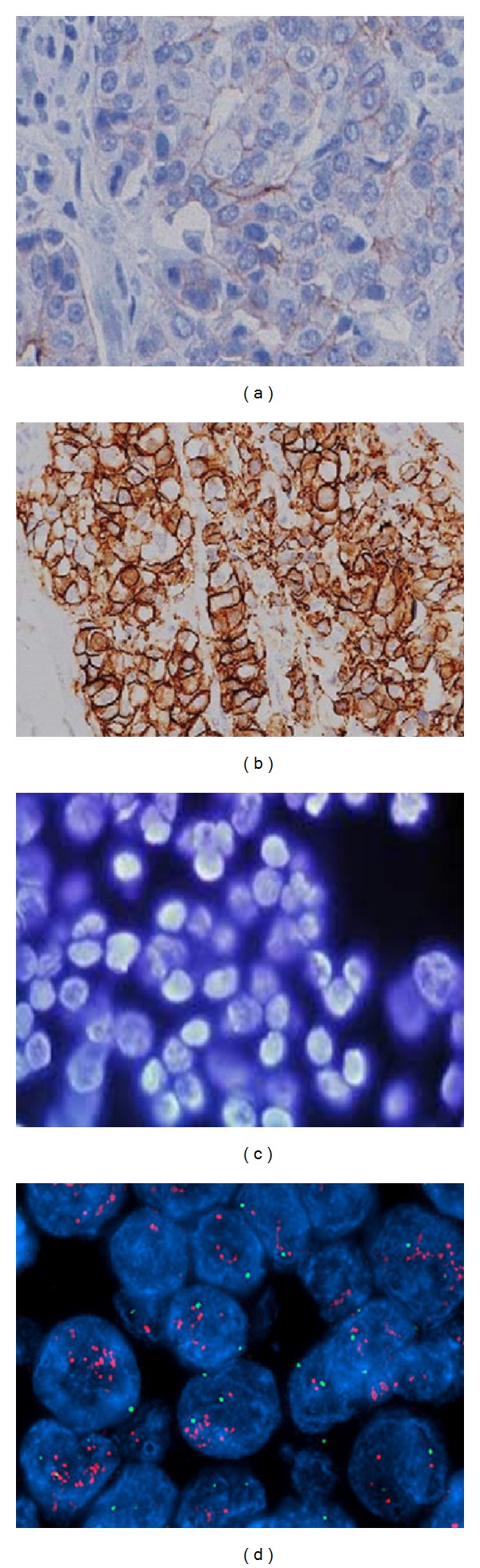
This image shows determination of HER2 status in 4 samples of breast tumor tissue. Samples (a) and (b) were analyzed by immunohistochemistry (IHC), while samples (c) and (d) were analyzed by fluorescence in situ hybridization (FISH). IHC detects protein and shows an increased expression of HER2 receptors, while FISH detects gene amplification. Sample (a) shows normal levels of HER2 protein expression, and sample (c) shows normal copy numbers of HER2 genes, while samples (b) and (d) show abnormal levels, respectively.

Figure 2 [59] shows HER2 analysis by IHC and FISH on breast tumor tissue.

*Gastric Cancer*. In gastric cancers, heterogeneity of the HER2 genotype can lead to discrepancies in the results from IHC and FISH testing [60]. Tumor heterogeneity was seen in roughly 4.8% of samples with moderate or strong HER2 staining and was higher than what was experienced in breast cancer (1.4%) [61]. ASCO/CAP guidelines state that intratumoral heterogeneity may contribute to HER2 testing inaccuracy. Incomplete basolateral membrane HER2 IHC staining is also more common in gastric cancer than in breast cancer. This is due to the higher frequency of glandular formations that occur in gastric tissue. In gastric tissue, the basolateral membrane is stained, not the luminal membrane resulting in the heterogeneity. Currently, there are no ASCO/CAP approved HER2 testing guidelines for gastric cancer.  Table 1 shows consensus panel recommendations on HER2 scoring for gastric/esophageal cancer [60, 61]. The National Comprehensive Cancer Network (NCCN) guidelines panel recommended that less than 3+ overexpression of HER2-neu by IHC should be additionally examined by FISH or other in situ hybridization methods. Gastric cancers with HER2 IHC overexpression of 3+ or FISH positive are considered positive and thus be treated with trastuzumab. Thus, HER2 3+ or FISH +/HER2 IHC 1+, FISH +/HER2 IHC 2+, FISH +/HER2 IHC 3+ gastric cancer patients should be treated with trastuzumab.

## 5. Targeting HER2

HER2 has been successfully targeted in breast cancer and gastric/gastroesophageal cancers. In ovarian cancer, HER2 is being investigated as a potential therapeutic target. There are several possible ways to target HER2.

### 5.1. Trastuzumab

Trastuzumab is a monoclonal antibody that binds to domain IV of the extracellular segment of the HER2 receptor. Proposed mechanisms of trastuzumab actions include (1) inhibition of HER2 shedding, (2) inhibition of PI3K-AKT pathway, (3) attenuation of cell signalling, (4) antibody-dependent cellular cytotoxicity, and (5) inhibition of tumor angiogenesis [62].

Trastuzumab was approved as part of a treatment regimen containing doxorubicin, cyclophosphamide, and paclitaxel for the adjuvant treatment of women with node-positive, HER2 overexpressing breast cancer. The approval was based on evidence of a significant prolongation in disease-free survival in women receiving trastuzumab and chemotherapy compared to those receiving chemotherapy alone.  Table 2 shows five pivotal trials involving more than 10,000 women which demonstrated that one year of trastuzumab therapy provided significant clinical benefit [63–66]. These trials demonstrated that inclusion of trastuzumab produces roughly a 50% improvement in disease-free survival and 33% improvement in overall survival, regardless of the chemotherapy regimen or sequence of trastuzumab delivery. In the metastatic HER2 breast cancer also, trastuzumab is recommended in the first-line setting. In a phase III trial, trastuzumab plus chemotherapy was associated with a significant improvement in time to disease progression, objective response rate, and 1-year survival compared with chemotherapy alone [67].

Trastuzumab was approved in combination with cisplatin and a fluoropyrimidine, for the treatment of patients with HER2 overexpressing metastatic gastric or gastroesophageal (GE) junction adenocarcinoma who have not received prior treatment for metastatic disease. The pivotal TOGA (trastuzumab for gastric cancer) trial demonstrated the median survival of 13.1 months for patients receiving trastuzumab and chemotherapy and 11.7 months for patients receiving chemotherapy alone. Trastuzumab was found to be most effective in prolonging survival in patients with HER2 IHC 3+ tumors as compared to patients with IHC 2+ tumors [68].

Trastuzumab is recommended at a dose of 4 mg/kg followed by 2 mg/kg weekly for breast cancer and 8 mg/kg followed by 6 mg/kg q3 weekly for gastric/gastroesophageal cancer. The duration of therapy is one year in adjuvant setting for breast cancer and till disease progression for metastatic breast, gastric, and gastroesophageal cancer. The most common adverse effects seen with trastuzumab are fever, vomiting, infusion reactions, diarrhea, headache, fatigue, rash, neutropenia, and anemia. The most serious adverse effects include cardiomyopathy, pulmonary toxicity, infusion reactions, and febrile neutropenia. Left ventricular ejection fraction (LVEF) should be evaluated in all patients prior to and during treatment with trastuzumab.

### 5.2. Lapatinib

Lapatinib is an orally active dual tyrosine kinase inhibitor which interrupts the HER2 and epidermal growth factor receptor (EGFR) pathways. Lapatinib is approved in combination therapy with capecitabine for HER2 overexpressing advanced and metastatic breast cancer patients who have received prior therapy including an anthracycline, a taxane, and trastuzumab. This was based on a study that demonstrated delay in time to disease progression when lapatinib was used in combination with capecitabine. The risk of disease progression was reduced by 51%, and the combination therapy was not associated with increases in toxic side effects [69]. Lapatinib is recommended at a dose of 1250 mg PO qDay on days 1–21 continuously in combination with capecitabine (2000 mg/m²/day PO divided q12hr) on days 1–14 in a repeating 21-day cycle.

Lapatinib is also approved in combination with leterozole for the treatment of postmenopausal women with hormone receptor and HER2 receptor positive metastatic breast cancers. The addition of lapatinib to letrozole is well tolerated and leads to a significantly greater progression free survival, overall response rate, and clinical benefit rate than with letrozole alone [70]. The most common adverse effects with lapatinib are diarrhea, anemia, hand-foot syndrome, liver dysfunction, nausea, rash, and neutropenia.

### 5.3. Pertuzumab

Pertuzumab is a humanized monoclonal antibody that blocks the activation of the HER2 receptor by hindering dimerization. Pertuzumab elicits action at a different ligand binding site from trastuzumab. It is approved in combination with trastuzumab and docetaxel in HER2-positive metastatic breast cancer patients previously not treated with hormone therapy or chemotherapy. The approval of pertuzumab was based on results from the Clinical Evaluation of Pertuzumab and Trastuzumab (CLEOPATRA) trial. The trial compared first-line trastuzumab plus docetaxel (plus placebo) to trastuzumab plus docetaxel plus pertuzumab in HER2-positive metastatic breast cancer. Results from the study showed an average increase in progression-free survival of 6.1 months in patients receiving pertuzumab in addition to trastuzumab and docetaxel with minimal to no increase in cardiac toxic effects [71]. Pertuzumab is also approved for use as neoadjuvant treatment in combination with trastuzumab and docetaxel for patients with HER2-positive, locally advanced, inflammatory, or early stage breast cancer. This was based on a randomized trial in which 39.3% of patients treated with pertuzumab, trastuzumab, and docetaxel achieved a pathologic complete response (pCR) compared with 21.5% of patients treated with trastuzumab and docetaxel at the time of surgery [72].

The recommended dose of pertuzumab is 840 mg initial dose followed by 420 mg every 3 weeks administered as an intravenous infusion over 30 to 60 minutes. The adverse effects seen with pertuzumab are alopecia, diarrhea, nausea, neutropenia, and cardiomyopathy.

### 5.4. Ado-Trastuzumab Emtansine

Ado-trastuzumab emtansine is an antibody-drug conjugate consisting of the monoclonal antibody trastuzumab linked to the cytotoxic agent mertansine (DM1). Most of the HER2-positive metastatic breast cancer patients eventually develop resistance. Ado-trastuzumab offers a novel mechanism for overcoming trastuzumab resistance by exploiting trastuzumab to target the cytotoxic activity of DM1 to HER2 overexpressing cells. Ado-trastuzumab is approved as a single agent for treatment of HER2-positive, metastatic breast cancer in patients who have already received trastuzumab and a taxane either separately or in combination. Approval was based on results from EMILIA trial which compared ado-trastuzumab to lapatinib plus capecitabine. The study showed a significantly prolonged progression-free survival and overall survival with less toxicity than lapatinib plus capecitabine [73].

The recommended dose of ado-trastuzumab is 3.6 mg/kg IV infusion q3 weeks until disease progression or unacceptable toxicity. The most common adverse effects include fatigue, nausea, musculoskeletal pain, thrombocytopenia, headache, transaminitis, constipation, and peripheral neuropathy. The serious adverse effects include liver failure, hepatic encephalopathy, nodular regenerative hyperplasia, cardiac dysfunction, and interstitial lung disease.

### 5.5. Neratinib

Neratinib is an oral irreversible tyrosine kinase inhibitor of HER2 and EGFR. A phase II open label study in locally advanced breast cancer (LABC) showed a 16-week progression-free survival rate of 75% in 36 trastuzumab-naive patients and 51% in previously treated disease [74]. Diarrhea was the most common grade 3/4 toxic effect (21%) in this study. Phase III evaluation of neratinib is ongoing in adjuvant trastuzumab-pretreated early-stage breast cancer.

### 5.6. Afatinib

Afatinib is an oral, irreversible inhibitor targeting EGFR/HER1, HER2, and HER4. Results from a phase II study of afatinib for HER2-positive MBC progressing posttrastuzumab (*N* = 41) showed 4 partial responses among 35 assessable patients. The most common all-grade treatment-related AEs included diarrhea (90.2%) and rash (65.9%) [75]. LUX-Breast 1 is an ongoing phase III study of vinorelbine plus either afatinib or trastuzumab for HER2-positive MBC in patients who failed one trastuzumab-containing regimen as first-line treatment of MBC or as adjuvant therapy [76].

## 6. Conclusion

HER2 has served as a prognostic and predictive biomarker in breast and gastric/gastroesophageal cancers. Therapies directed against HER2 have revolutionized the treatment of HER2 overexpressing breast and gastric cancers and improved the clinical outcome. Although HER2 overexpression was also found to correlate with poor outcome in other cancers, HER2 directed therapies provided disappointed results. Various novel HER2 directed agents alone or in combination are under investigation and in near future we will be expecting more varied implications of HER2 directed therapies. Till more robust data on the prognostic significance of HER2 in other cancers is available, HER2 testing and HER2 directed therapies are recommended in only breast and gastric/gastroesophageal cancers.

## Figures and Tables

**Table 1:** Consensus panel recommendations for HER2 scoring in gastric/esophageal cancer.

Score	Specimen	HER2 overexpression assessment
0	No reactivity or membranous reactivity in <10% of cells (resection): in biopsies only one cohesive cluster of >5 cells is required.	Negative
1+	Faint membranous reactivity in >10% of tumor cells (resection): in biopsies only one cohesive cluster of >5 cells is required.	Negative
2+	Weak to moderate incomplete (basolateral) membranous staining in >10% of tumor cells (resection): in biopsies only one cohesive cluster of >5 cells is required.	Equivocal
3+	Moderate to strong incomplete (basolateral) membranous staining in >10% of tumor cells (resection): in biopsies only one cohesive cluster of >5 cells is required.	Positive

**Table 2:** Five pivotal trials for adjuvant trastuzumab in breast cancer.

Study	Control arm	Trastuzumab arm	Reduction in relative risk of recurrence	DFS hazard ratio
NSABP B-31 (*N* = 2700)	AC → T	AC → TH	Joint analysis 52%	0.48
NCCTG N9831 (*N* = 3300)	AC → T	AC → TH		
HERA (*N* = 5090)	Any	Trastuzumab 1 year	46%	0.54
BCIRG 006 (*N* = 3150)	AC → D	AC → DH DCH	40% 33%	0.49 0.61
FINHer (*N* = 232)	D → FEC V → FEC	DH → FEC VH → FEC		0.42

NSABP, National Surgical Adjuvant Breast and Bowel Project; NCCTG, North Central Cancer Treatment Group; HERA, Herceptin Adjuvant Trial; BCIRG, Breast Cancer International Research Group; FINHer, Finland Herceptin Study. A, doxorubicin; C, cyclophosphamide; D, docetaxel; E, epirubicin; F, fluorouracil; H, trastuzumab; T, paclitaxel; V, vinorelbine; DFS, disease-free survival.
